# Orexin-1 Receptor Co-Localizes with Pancreatic Hormones in Islet Cells and Modulates the Outcome of Streptozotocin-Induced Diabetes Mellitus

**DOI:** 10.1371/journal.pone.0008587

**Published:** 2010-01-06

**Authors:** Ernest Adeghate, Maria Fernandez-Cabezudo, Rashed Hameed, Hussain El-Hasasna, Mohamed El Wasila, Tariq Abbas, Basel al-Ramadi

**Affiliations:** 1 Department of Anatomy, Faculty of Medicine and Health Sciences, United Arab Emirates University, Al Ain, United Arab Emirates; 2 Department of Biochemisty, Faculty of Medicine and Health Sciences, United Arab Emirates University, Al Ain, United Arab Emirates; 3 Department of Medical Microbiology, Faculty of Medicine and Health Sciences, United Arab Emirates University, Al Ain, United Arab Emirates; 4 Department of Pharmacology, Faculty of Medicine and Health Sciences, United Arab Emirates University, Al Ain, United Arab Emirates; 5 Veterinary Laboratory, Department of Agriculture, Al Ain, United Arab Emirates; University of Bremen, Germany

## Abstract

Recent studies have shown that orexins play a critical role in the regulation of sleep/wake states, feeding behaviour, and reward processes. The exocrine and endocrine pancreas are involved in the regulation of food metabolism and energy balance. This function is deranged in diabetes mellitus. This study examined the pattern of distribution of orexin-1 receptor (OX_1_R) in the endocrine cells of the pancreas of normal and diabetic Wistar (a model of type 1 diabetes), Goto-Kakizaki (GK, a model of type 2 diabetes) rats and in orexin-deficient (OX^−/−^) and wild type mice. Diabetes mellitus (DM) was induced in Wistar rats and mice by streptozotocin (STZ). At different time points (12 h, 24 h, 4 weeks, 8 months and 15 months) after the induction of DM, pancreatic fragments of normal and diabetic rats were processed for immunohistochemistry and Western blotting. OX_1_R-immunoreactive nerves were observed in the pancreas of normal and diabetic Wistar rats. OX_1_R was also discernible in the pancreatic islets of normal and diabetic Wistar and GK rats, and wild type mice. OX_1_R co-localized with insulin (INS) and glucagon (GLU) in the pancreas of Wistar and GK rats. The number of OX_1_R-positive cells in the islets increased markedly (p<0.0001) after the onset of DM. The increase in the number of OX_1_R-positive cells is associated with a high degree of co-localization with GLU. The number of GLU- positive cells expressing OX_1_R was significantly (p<0.0001) higher after the onset of DM. The tissue level of OX_1_R protein increased with the duration of DM especially in type 1 diabetes where it co-localized with cleaved caspase 3 in islet cells. In comparison to STZ-treated wild type mice, STZ-treated OX^−/−^ animals exhibited reduced hyperglycemia and handled glucose more efficiently in glucose tolerance test. The findings suggest an important role for the OX-OX_1_R pathway in STZ-induced experimental diabetes.

## Introduction

Recent studies have shown that orexins A and B play a critical role in the regulation of sleep/wake states, feeding behaviour, and reward processes [1], [2]. They also participate in the regulation of food intake in a dose-dependent fashion [3], [4], [5] and consequently of energy balance [6]. Orexins are related to hypocretins and were first described by Sakurai et al. [7]. Orexins have been demonstrated in the lateral hypothalamic region, an area of the brain closely associated with the regulation of behaviour [8]. A strong indication of the role of orexin-positive neurons in feeding is that genetic ablation of orexigenic neurons results in narcolepsy, hyperphagia and obesity [9]. Orexin-1 receptor (OX_1_R) was the first of the two orexin receptors to be discovered and binds orexin-A with a much higher affinity compared to orexin B. The human OX_1_R protein consists of 425 amino acids and seven putative transmembrane helices [10]. OX_2_R consists of 444 amino acids and has equal affinities for both orexin A and B ligands. OX_2_R shares about 64% amino acid identity with OX_1_R [1]. In addition, OX_1_R is more selective for orexin A [11], [12] and usually coupled to the Gq subclass of G proteins while OX_2_R can couple to either Gq and/or Gi/o subset of G proteins [1]. The binding of OX_1_R and OX_2_R to either orexin A or B causes intracellular influx of Ca^2+^ resulting in increased concentration of intracellular Ca^2+^ [11].

OX_1_R and OX_2_R are present in the lateral hypothalamic area (LHA), which is rich in orexin-positive nerves [13]. In the hypothalamic region, OX_1_R mRNA is located mainly in the dorsomedial area of the ventromedial hypothalamic nucleus (VMH) and the anterior hypothalamic territory, whereas, OX_2_R mRNA is observed in several hypothalamic nuclei including the arcuate nucleus, posteroventricular nucleus, LHA, and tuberomammilary bodies [13]. The localization of orexin receptors to these hypothalamic nuclei supports the purported function of orexins on the regulation of feeding, since food intake and appetite are regulated by these cortical regions (VMH, LHA, arcuate nucleus). The role of VMH in the regulation of food intake is very strong because the selective ablation of VMH results in obesity [12], [14]. Moreover, expression of OX_1_R mRNA has been demonstrated in many other parts of the brain, including the hippocampus, locus coeruleus and raphe nuclei [13]. The localization of OX_1_R mRNA in these cortical regions, which has large quantities of adrenergic and serotonergic neurotransmitters suggests that orexins may also play an important role in the regulation of serotonergic as well as adrenergic systems. OX_2_R is also expressed in the amygdala [13] a brain region that is strongly associated with memory and emotion. OX_1_R has also been shown in the spinal cord and sensory ganglion in the mouse [15].

The expression of orexin receptor mRNA is not confined only to the central nervous system. Low OX_1_R mRNA expression has been observed in the adrenal gland of the rat [16] and human [17], stomach and intestinal circular muscle as well as mucosal and myenteric nerve plexuses in rat [18] and in the bovine urethroprostatic complex [19]. In contrast, OX_2_R mRNA expression is localized to neuroendocrine cells of the alimentary mucosa [20]. The pancreas has been shown to particularly express OX_1_R mRNA [12]. Previous studies from our laboratory have shown that OX_1_R is present in nerves and endocrine cells of the pancreas of normal and diabetic rats [21].

Since diabetes mellitus is associated with a dysfunction of glucose metabolism, the aim of this study was to examine whether diabetes mellitus will induce changes in the pattern of distribution of OX_1_R in the endocrine cells of Wistar and GK rats and mice pancreas.

## Materials and Methods

### Experimental Animals

#### a). Streptozotocin-induced diabetes mellitus (a model of type 1 diabetes)

Male Wistar rats (12-week old) obtained from United Arab Emirates University breeding colony, weighing approximately 250 g were used in this study. The study was approved by the Animal Ethics Research Committee at the Faculty of Medicine & Health Sciences. The guidelines set by this committee for animal husbandry and welfare, based on Helsinki Declaration of 2006 was followed. The rats were divided into two groups, streptozotocin (STZ)-induced diabetics and age-matched controls. Diabetes was induced by a single intraperitoneal injection of STZ (Sigma, Poole, UK) at 60 mg kg^−1^ (200 mg kg^−1^ for mice) prepared in 5 mM citrate buffer pH 4.50 [22]. The animals were kept in plastic cages and maintained on standard laboratory animal diet with food and water *ad libitum*. One-Touch II ® Glucometer (LifeScan, Johnson and Johnson, Milpitas, CA, USA) was used to measure the blood glucose for each individual animal. The animals were considered diabetic if the random blood glucose levels were ≥300 mg dl^−1^. At different time points (12 h, 24 h, 4 weeks, 8 months and 15 months) after the induction of DM, all of the animals from both groups were sacrificed under chloral hydrate general anesthesia (7% chloral hydrate 6-ml kg^−1^ of body weight, given intraperitoneally). The tail end of the pancreas was rapidly removed, fixed in Zamboni's solution [23] and processed for immunohistochemistry and immunofluorescence. Pancreatic tissue samples were also expeditiously removed, frozen and set aside for Western blot analysis. Orexin-deficient and C57BL/6 wild type mice were treated with STZ and processed in a similar way after 1 week.

#### b). Goto-Kakizaki rats (a model of type 2 diabetes mellitus)

The colony of GK rats were obtained from Taconic Inc. (Ejby, Denmark) and bred in our laboratory. GK rats are non-obese and exhibits similar metabolic, hormonal and vascular disorders to the human disease [24]. They are known to have relatively mild diabetes until the pancreas is exhausted [24]. Six GK rats were used for the experiment.

#### c). Orexin deficient (OX^−/−^) mice

Founder orexin deficient (OX^−/−^) mice were on a C57BL/6J-129/SvEV background, and their offspring were backcrossed with C57BL/6J mice for 6–8 generations [25]. OX^−/−^ mice were kindly donated by Dr. Thomas Scammell, Harvard University Medical School, Boston, USA. Five OX^−/−^ and 6 wild type mice were used for the experiment.

### Immunohistochemistry

Pancreatic tissue fragments from six normal and diabetic rats from each group (time point), were trimmed free of adherent fat and connective tissue and cut into small pieces (2 mm^3^) and fixed overnight in Zamboni's solution [23], dehydrated and embedded in paraffin wax according to a previously described method [22]. Sections of 6 µm thickness were cut on a microtome (Shandon AS325, Pittsburg, PA, USA) and processed for immunohistochemistry according the method described by Adeghate et al. [21], [22]. Briefly, after 30 min incubation in the blocking reagent the appropriate dilution of primary antibodies (OX_1_R) and negative control (Phosphate buffered solution: PBS) reagents were applied. The sections were incubated in primary antibodies for 24 h at 4°C. The slides were then washed and incubated for 30 min with prediluted biotinylated anti-rabbit IgG. After washing in PBS the sections were incubated in streptavidin peroxidase conjugate for 45 min. After a final wash in PBS, the peroxidase activity was revealed by incubating the specimens in 3, 3-diaminobenzidine tetrahydrochloride containing 0.03% hydrogen peroxide in PBS. The slides were later washed, and counter-stained with haematoxylin for 30 s and mounted in Cytoseal 60 (Stephens Scientific, Riverdale, USA). Slides were examined under Zeiss Axiophot microscope and immunopositive islets cells and nerves of the tissue sections were photographed. The antiserum to OX_1_R was purchased from Santa Cruz (CA, USA) and was used at a dilution of 1∶1000. No specific immunostaining was observed in pancreatic tissue when primary antiserum was omitted.

### Morphometric analysis of OX_1_R-, insulin-, glucagon- and caspase-immunoreactive cells in pancreatic endocrine cells

In order to determine whether diabetes mellitus influences the number and pattern of distribution of OX_1_R-, insulin-, glucagon- and caspase-immunoreactive cells, the total number of cells in the islets of Langerhans of normal and diabetic rats was counted using Axiovision® Image Analysis System (Zeiss, Gottingen, Germany) attached to a fluorescent microscope. Thereafter, OX_1_R-immunoreactive cells and those co-localized with either insulin (INS) or glucagon (GLU) in a given islet were also counted. The number of OX_1_R-immunoreactive cells or those that co-localized with INS or GLU was divided by the total number of cells to give the percentage distribution. Isolated OX_1_R-postive cells or those co-localized with INS- or GLU-positive cells were counted in visible sections at X40 magnification. A total of 10 random islets were taken from a total of 6 slides for each group. The values obtained from sections of the islets of Langerhans of normal rat were compared with those of diabetic rat.

### Immunofluorescence

#### a). Co-localization of OX_1_R with pancreatic hormones in islet cells

The co-localization of OX_1_R with either INS or GLU in pancreatic islets of normal and diabetic rats was performed with double-labeled immunofluorescence method as described by Adeghate and Ponery [26]. Briefly, 6 µm thick sections of normal and diabetic rat pancreas were deparaffinized with xylene, and rehydrated with descending concentrations of ethanol. The sections were treated with a blocking agent for 30 min at room temperature after washing in PBS. The sections were later incubated with sheep OX_1_R goat polyclonal antibody (1∶1000) overnight at 4°C. The same sections were later incubated overnight at 4°C with either INS or GLU antibody (Prediluted from Dako, Denmark) after labeling with TRITC (1: 100). Sites of immunoreaction were detected using anti-guinea pig FITC (1: 100) for INS and anti-rabbit FITC for GLU. The sections were mounted in Immunomount® (Shandon, Pittsburgh, PA, USA) and viewed with a Zeiss fluorescent microscope.

#### b). Co-localization of OX_1_R with Caspase 3 in islet cells

Some studies [27], [28], [29] have linked OX_1_R with the apoptotic pathway, where orexin stimulates OX_1_R to induce large scale cell death in cancer cell lines. In order to determine whether there is a link between OX_1_R and the apoptotic pathway in pancreatic islet cells, a double staining immunofluorescence method described above was used to identify OX_1_R and cleaved caspase 3 (pre-diluted; an apoptotic marker; Cell Signaling Technology, Inc., Beverly, MA, USA; Cat # 8120) simultaneously in pancreatic islets of OX^−/−^ and C57BL/6 mice.

### Western blotting

#### a). The time course for the development of increased OX_1_R

Pancreatic tissue samples from different experimental time points were homogenized in 1 ml of lysis buffer [1% NP-40, 25 mM Tris pH 7.4, 0.1 mM EDTA, 1 mM DTT, protease inhibitor cocktail set III (Calbiochem, San Diego, CA, USA) on ice. After incubating the homogenates on ice for 15 min, samples were centrifuged at 13000 rpm for 15 min at 4°C. Clarified cell lysates were collected and total protein concentrations were measured by the Bradford method (Bio-Rad protein assay, Bio-Rad laboratories, Munich, Germany). The lysates (200 µg protein/lane) were resolved on a 10% SDS-PAGE, transferred to a PVDF membrane (Bio-Rad), and blocked with 5% nonfat milk powder in TBS containing 0.01% Tween 20 at room temperature for 1 h. Membranes were incubated with OX_1_R antibody (1∶1000, goat-anti-rat orexin R-1, Santa Cruz Biotechnology, USA) overnight at 4°C, washed, and incubated with horse radish peroxidase-conjugated anti-goat IgG secondary antibody (Sigma, UK) for 1.5 h at room temperature. Blots were washed and developed using SuperSignal substrate (Pierce, Rockford, IL, USA).

#### b). OX_1_R and its relation to apoptosis

In a bid to examine a possible link between OX_1_R and apoptosis, pancreatic tissue samples from control OX^−/−^ and C57BL/6 and STZ-treated OX^−/−^ and C57BL/6 mice were homogenized and processed for Western blotting as described above. Membranes were incubated overnight at 4°C with either anti orexin R-1 or anti β-actin antibodies (1∶1000, Santa Cruz Biotechnology, USA), or anti-PARP antibody (1∶1000, R&D Systems Inc., Minneapolis, USA). The blots were further processed as described above and developed using SuperSignal substrate.

### Glucose tolerance test

In order to investigate the ability of orexin knockout (OX^−/−^) and wild type (C57BL/6) mice to handle glucose challenge, control OX^−/−^ and C57BL/6 and STZ-treated OX^−/−^ and C57BL/6 mice were fasted overnight (12 h) prior to the test. Each mouse was given a glucose load of 3 g/kg body weight intra-peritoneally, according to Zhang and Tan [30]. Blood samples were collected from the tail vein at time 0 (prior to the glucose load), 30, 60 and 180 minutes after the glucose load.

### Statistical analysis

All values were expressed as mean±standard error of the mean (SEM). Statistical significance was assessed using Student's *t*-test. Values with p<0.05 were accepted as significant.

## Results

### OX_1_R in nerves innervating the pancreas

**Figure 1:**
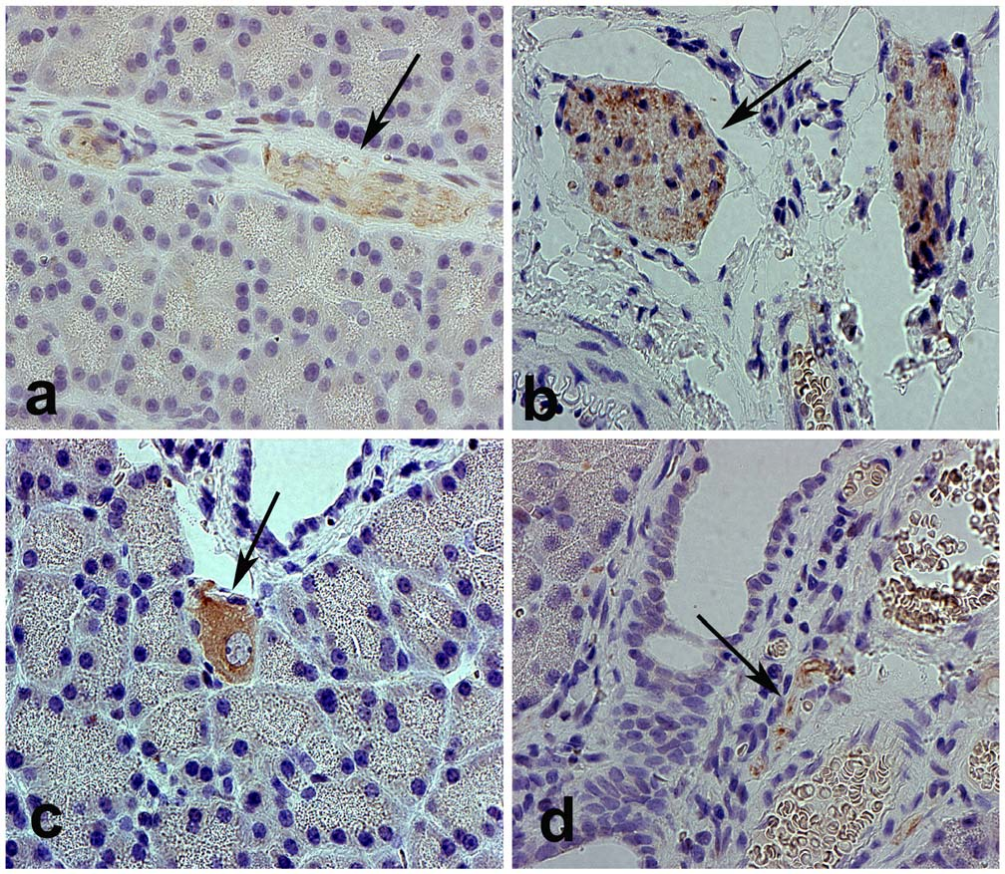
Immunolocalization of OX_1_R in intrapancreatic nerves. Light micrographs showing OX_1_R-immunoreactive nerve fibers (arrow) in the pancreas of normal (a), and diabetic (b, d) rats. OX_1_R expression is equally present in the nerve fibers of both normal and diabetic (4 weeks after the onset of diabetes) rats. Ganglion cells (arrow) of normal pancreas (c) expressed OX_1_R. Magnification: ×400.

Peripheral nerves located in the interlobular, perivascular, and periductal regions of the pancreas of both normal and diabetic rats contain OX_1_R (Fig. 1a, b, c, d). Moreover, intrinsic, neuronal ganglion cells of the pancreas of normal rat also expressed OX_1_R (Fig. 1c).

### Co-localization of OX_1_R with insulin (INS) and glucagon (GLU)

**Figure 2:**
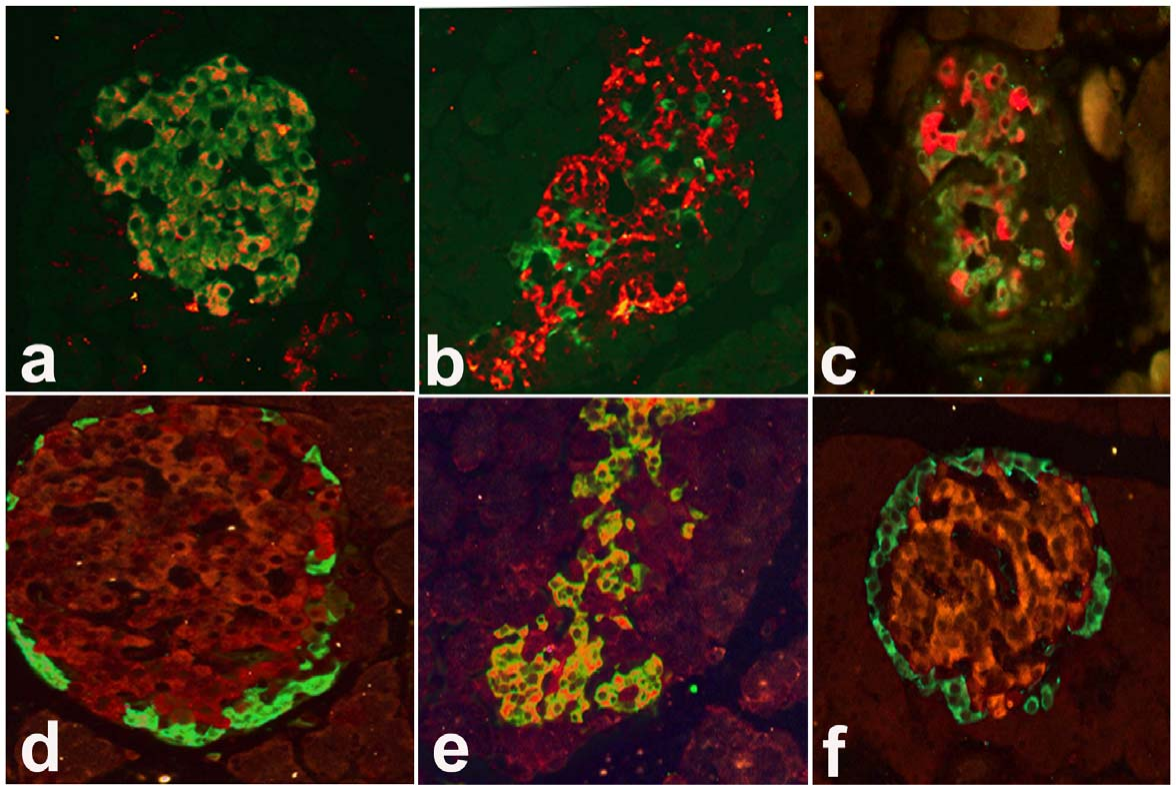
Co-localization of orexin-1 receptor (OX_1_R) with either insulin (INS) or glucagon (GLU) in pancreatic islets. Immunofluorescence images showing orexin-1 receptor (OX_1_R)-immunoreactive cells (red) with either, (a) INS (green) or (d) GLU (green) in the pancreatic islet of normal rats. Many cells (orange-yellow) contain both OX_1_R and INS in the pancreas of normal Wistar rats (a). Only few cells contain both OX_1_R and GLU in the endocrine pancreas of normal Wistar rats (d). In diabetic (4 weeks after the onset of diabetes) rats, the number of INS cells decreased (b). However, some surviving INS-positive cells also contained OX_1_R (orange-yellow) (b). OX_1_R also co-localized (orange-yellow) with INS in the pancreatic islets of GK rats (c). A large number of GLU-positive cells expressed OX_1_R after the onset of streptozotocin-induced diabetes (e). There was little to no co-localization of GLU and OX_1_R in the islet of GK rats (f). INS = insulin, GLU = glucagon; GK = Goto Kakizaki. Magnification: ×200

Figure 2 shows the expression of OX_1_R and the peptide hormones, INS and GLU in the islet cells of the pancreas of normal and diabetic Wistar and GK rats. Islet cells expressing OX_1_R were discernible in both the peripheral and central regions of the islets of Langerhans in normal Wistar rats (Fig. 2a, 2d). The pancreas of Wistar diabetic (a model of type 1 diabetes) and GK (a model of type 2 diabetes) rats also contained OX_1_R. OX_1_R immunoreactive cells were located in the peripheral as well as in the central parts of the islets. The number of OX_1_R-immunoreactive cells in the pancreatic islet of normal and diabetic rats was 21.24±4.5 and 62.8±10.5, respectively.

Figure 2a showed INS and OX_1_R-positive cells in pancreatic islets of normal rats. OX_1_R was expressed in many INS-positive cells in the pancreas of normal rat. However, in diabetic rat pancreas (Fig. 2b), only a few cells contained insulin. Few of the surviving INS-positive cells expressed OX_1_R as well. In a similar fashion, INS-producing cells in the pancreas of GK rats also contained OX_1_R (Fig. 2c).

In contrast to the small degree of co-localization observed between OX_1_R and INS pancreatic islet cells of diabetic Wistar rats, a much larger subset of GLU-positive cells expressed OX_1_R in diabetic Wistar rats (Fig. 2e) compared to normal pancreas (Fig. 2d). The number of GLU-immunoreactive cells increased significantly after the onset of diabetes. This increase was associated with a concomitant increase in the number of cells expressing OX_1_R (Fig. 2e). GLU-immunoreactive cells expressing OX_1_R were observed in both the peripheral and central portions of the islet after the onset of diabetes. The expression of OX_1_R in GLU-positive cells was significantly higher in diabetic rats compared to normal. The degree of co-localization of between GLU and OX_1_R in GK rats (Fig. 2f) was not significantly different from that observed in Wistar normal (Figs 2d).

### Morphometric analysis of OX_1_R immunoreactive cells co-localized with either insulin (INS) or glucagon (GLU) in Wistar rats

**Figure 3:**
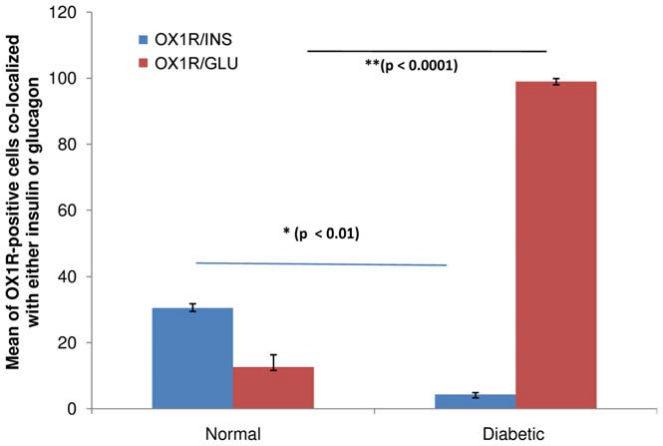
Mean number of pancreatic OX_1_R-immunoreactive cells containing either insulin or glucagon. Histograms of the mean of the distribution of OX_1_R-immunoreactive cells containing either INS or GLU in normal and diabetic (4 weeks after the onset of diabetes) rat pancreas. *(p<0.01: OX_1_R/INS in control versus diabetic); **(p<0.0001: OX_1_R/GLU in control versus diabetic). INS = insulin, GLU = glucagon. (10 islets from 6 animals/group).

OX_1_R co-localized with INS in a large number of INS-producing beta cells in the pancreas of normal Wistar rat. The number of OX_1_R-containing cells that contain INS decreased significantly (p<0.001) after the onset of diabetes. Although some OX_1_R-positive cells also contained GLU, the percentage distribution of GLU-immunoreactive cells that co-localized with OX_1_R rose significantly (p<0.0001) after the onset of diabetes when compared to normal (Fig. 3).

### OX_1_R immunoreactive cells in the islet of Goto Kakizaki rats

**Figure 4:**
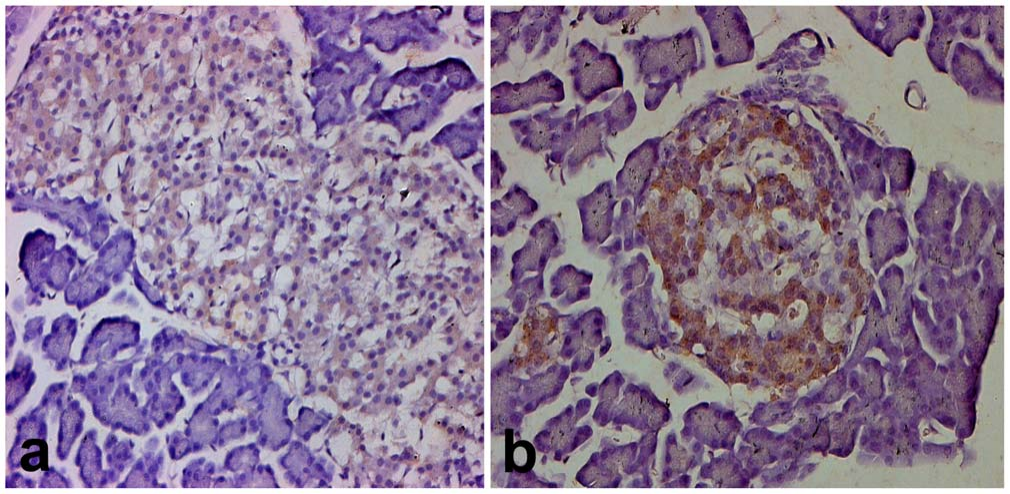
Comparison of OX_1_R-immunoreactivity in the endocrine pancreas of Wistar and GK rats. Light micrographs showing OX_1_R-immunoreactive cells in the pancreatic islet of normal Wistar (a) and Goto Kakizaki (b) rats. Note that the islet cells of Goto Kakizaki stains more intensely for OX_1_R compared to Wistar. Magnification: ×200.

The Goto-Kakizaki (GK) rat is a model of type 2 diabetes with similar metabolic disorders compared to human diabetes. GK rats are hyperglycaemic and have an impaired insulin secretion. Immunohistochemistry was performed to examine whether the pattern of distribution of OX_1_R is similar in the pancreatic islets of GK compared to Wistar rats. The pancreatic islets of GK rats contain numerous OX_1_R-imunoreactive cells similar to that observed in the islet of diabetic Wistar rats (Fig. 4).

### Time course of diabetes-induced changes in OX_1_R expression

**Figure 5:**
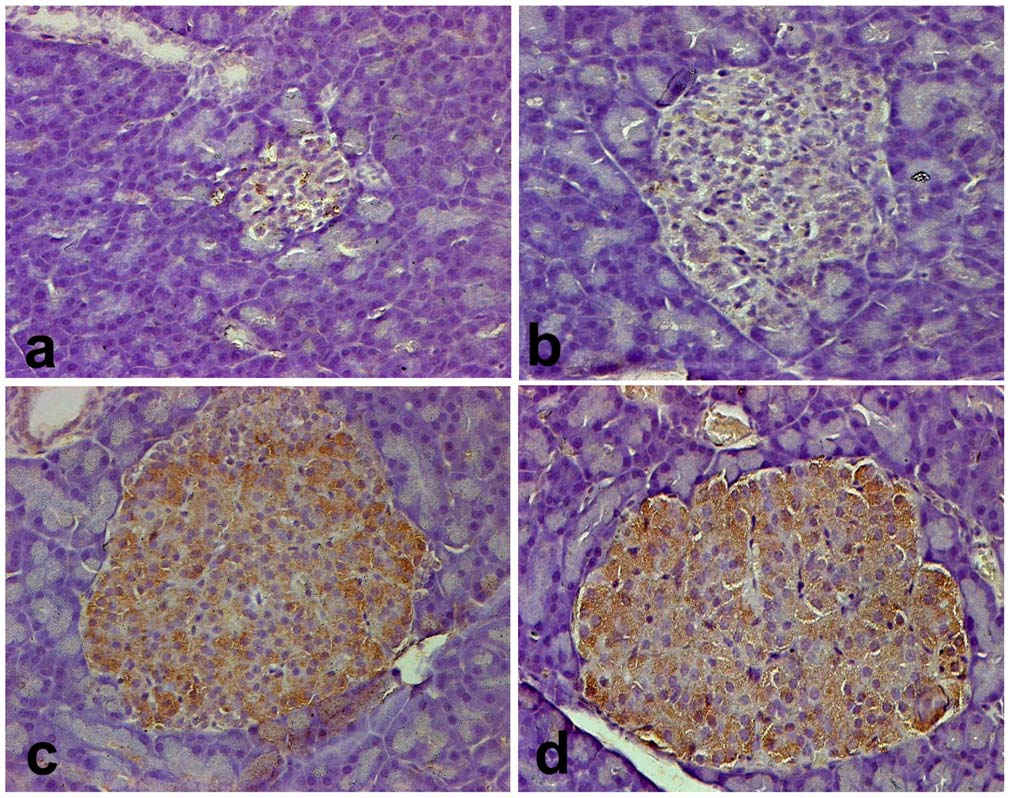
Localization of OX_1_R in the pancreatic islets of Wistar rats with acute, short- and long-term diabetes mellitus. Light micrographs showing OX_1_R-immunoreactive cells in the pancreatic islet of Wistar 12 h (a); 24 h (b); 8 months (c) and 15 months (d) after the onset of diabetes. Note the large number of islet cells containing OX_1_R in long-term diabetes (c) and (d). Magnification: ×200.

The expression of OX_1_R in the pancreas of diabetic rats was investigated at different time points after the induction of DM. The expression of OX_1_R increased with the duration of DM from pale staining 12–24 h after the onset of DM to intense expression 8–15 months after the induction of DM (Fig. 5).

### Semi-quantitative analysis of OX_1_R protein expression in pancreatic islets of normal and diabetic Wistar rats

**Figure 6:**
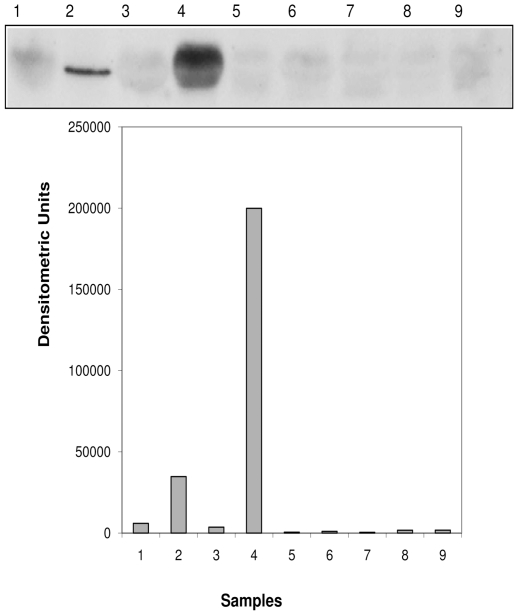
Expression of orexin-1 receptor (OX_1_R) protein in the pancreatic islets of Wistar rats with acute, short- and long-term diabetes mellitus. Western blot analysis of OX_1_R protein expression in the pancreas of normal and diabetic rats at different time points of diabetes. Note that the tissue level of OX_1_R protein was significantly (p < 0.0001, Students'*t*-test) higher in the pancreas of long-term (8 and 15 months) rats compared to that of rats with acute and short-term diabetes mellitus (n = 6). 1: Control 8 months; 2: Diabetic 8 months; 3: Control 15 months; 4: Diabetic 15 months 5, 6 and 7: Diabetic 12 h; 9 Diabetic 24 h.

Western blot analysis of pancreas segments from normal and diabetic rats was performed to quantify the tissue content of OX_1_R. The expression of OX_1_R protein was highest in the pancreas of rats with long-term DM, namely 8 and 15 months and low in the pancreas of rats with acute to short-term (12–24 h) DM (Fig. 6).

### OX_1_R and markers of apoptosis

#### a). Co-localization of OX_1_R and Caspase 3

**Figure 7:**
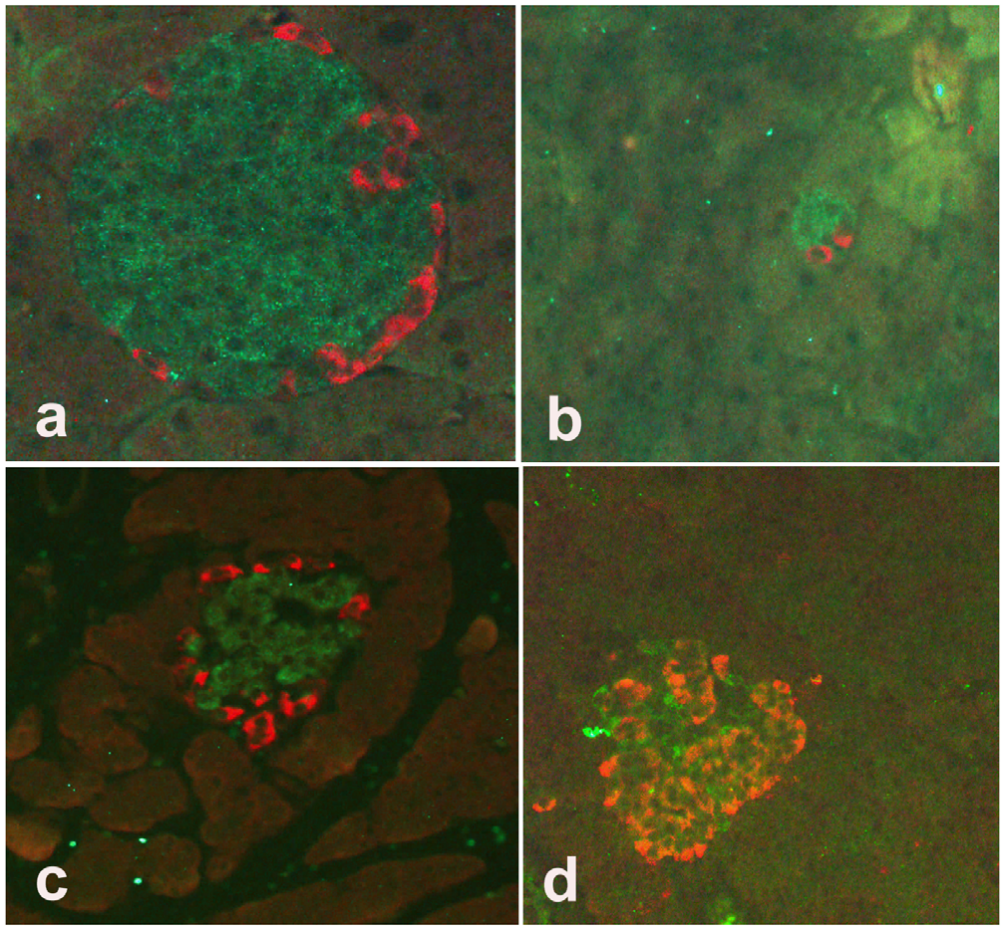
Co-localization of orexin-1 receptor (OX_1_R) with cleaved caspase 3 in pancreatic islet cells of wild type (C57BL/6) mice, orexin knockout mice, and normal and diabetic Wistar rats. Immunofluorescence images showing co-localization of orexin-1 receptor (OX_1_R) (green) with cleaved caspase 3 (red) in pancreatic islet cells of C57BL/6 mice (a), orexin knockout mice (b), normal Wistar (c) and diabetic Wistar (d) rats. OX_1_R-positive cells are located in the peripheral region of the islets of C57BL/6 and orexin knockout mice and normal Wistar rats. However, in diabetic Wistar rats (d), there is a high degree of co-localization (orange-yellow) between OX_1_R and caspase 3 in many pancreatic islet cells. It is worth noting that the number of OX_1_R (green) and cleaved caspase 3 (red) immunoreactive cells are significantly reduced in orexin knockout mice. Magnification: ×200.

In order to examine a possible link between OX_1_R and cleaved caspase 3 in pancreatic islet of OX^−/−^ and C57BL/6, we used immunofluorescence method to simultaneously localize OX_1_R and cleaved caspase 3. Cleaved caspase 3-positive cells were located in the peripheral region of pancreatic islets in OX^−/−^, C57BL/6 mice and normal Wistar rats (Figs. 7a–c). The pancreatic islet of C57BL/6 mice contained a significantly larger number of OX_1_R-immunoreactive cells compared to the mutant type. The ratio of OX_1_R-positive cells in the pancreas of the wild type to that of OX^−/−^ mice was 20∶1. In addition, the number of cleaved caspase 3-positive cells was significantly lower in OX^−/−^ mice when compared to that of wild type. The ratio of cleaved caspase 3-positive cells in the islets of Langerhans of the wild type to that of orexin knockout mice was 6∶1 (Fig. 7).

**Figure 8:**
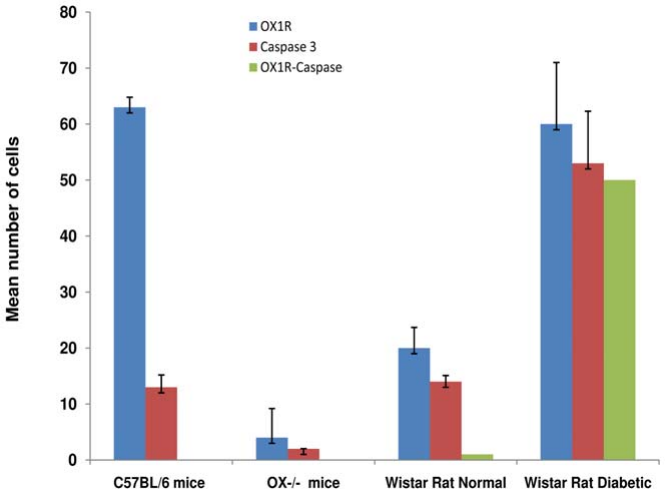
Distribution of OX_1_R-, cleaved caspase 3- and OX_1_R/cleaved caspase 3- immunopositive cells in pancreatic islet of C57BL/6 mice, orexin knockout mice, normal and diabetic Wistar rats. Histograms of the pattern of distribution of OX_1_R-positive (green), cleaved caspase 3-immunoreactive cells (red) and cells containing both OX_1_R and cleaved caspase 3- in the pancreas of in pancreatic islet cells of C57BL/6 mice, orexin knockout mice, normal Wistar and diabetic Wistar rats. Note the direct correlation between the number of OX_1_R-positive cells and that of cleaved caspase 3. The number of cells containing both OX_1_R cleaved caspase 3 is significantly elevated in streptozotocin-induced diabetic Wistar rats.

Cleaved caspase 3-positive cells were relatively high in the islets of normal Wistar rats compared to wild type and OX^−/−^ mice. However, the number of OX_1_R- and cleaved caspase 3-immunoreactive cells increased significantly (p<0.001) after the onset of STZ-induced diabetes. The degree of co-localization of OX_1_R with cleaved caspase 3 was more than 90%. This values was significantly higher in Wistar diabetic rats than those obtained in orexin deficient or wild type mice (Fig. 8).

#### b). Western blot analysis of OX_1_R and the apoptotic marker PARP {poly (ADP-ribose) polymerase} in pancreatic islets of control or STZ-treated OX^−/−^ and C57BL/6 mice

To further elucidate the link between OX_1_R and apoptosis, we used Western blotting technique to semi-quantify pancreatic tissue levels of OX_1_R protein and the apoptotic marker PARP polymerase, a DNA repair enzyme which is targeted by active caspase 3 during apoptosis.

**Figure 9:**
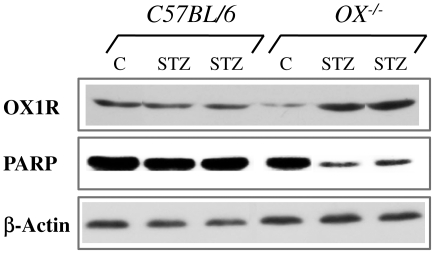
Expression of OX_1_R protein and PARP (poly-ADP-ribosome polymerase) in the pancreas of control or streptozotocin (STZ)-treated OX^−/−^ and C57BL/6 mice. Western blot analysis of OX_1_R protein and PARP (poly-ADP-ribosome polymerase) expression in the pancreas of control or STZ-treated OX^−/−^ and C57BL/6 mice are shown in lanes 1 and 2. Lane 1 shows that the OX_1_R expression was not enhanced in the pancreas of wild type mice but was upregulated in the pancreas of OX^−/−^ mice after the onset of STZ-induced diabetes. In contrast, the expression of PARP was robust in the pancreas of C57BL/6 mice but decreased markedly in OX^−/−^ mice treated with STZ (Lane 2). Lane 3 shows the expression of the control protein, beta actin.

OX_1_R expression was not enhanced in the pancreas of wild type mice but was upregulated in the pancreas of OX^−/−^ mice after the onset of STZ-induced diabetes. In contrast, the expression of PARP, an indication of proteolysis by active caspase 3, was robust in the pancreas of C57BL/6 mice but decreased markedly in OX^−/−^ mice (Fig. 9).

### Glucose handling in control or STZ-treated OX^−/−^ and C57BL/6 mice

**Figure 10:**
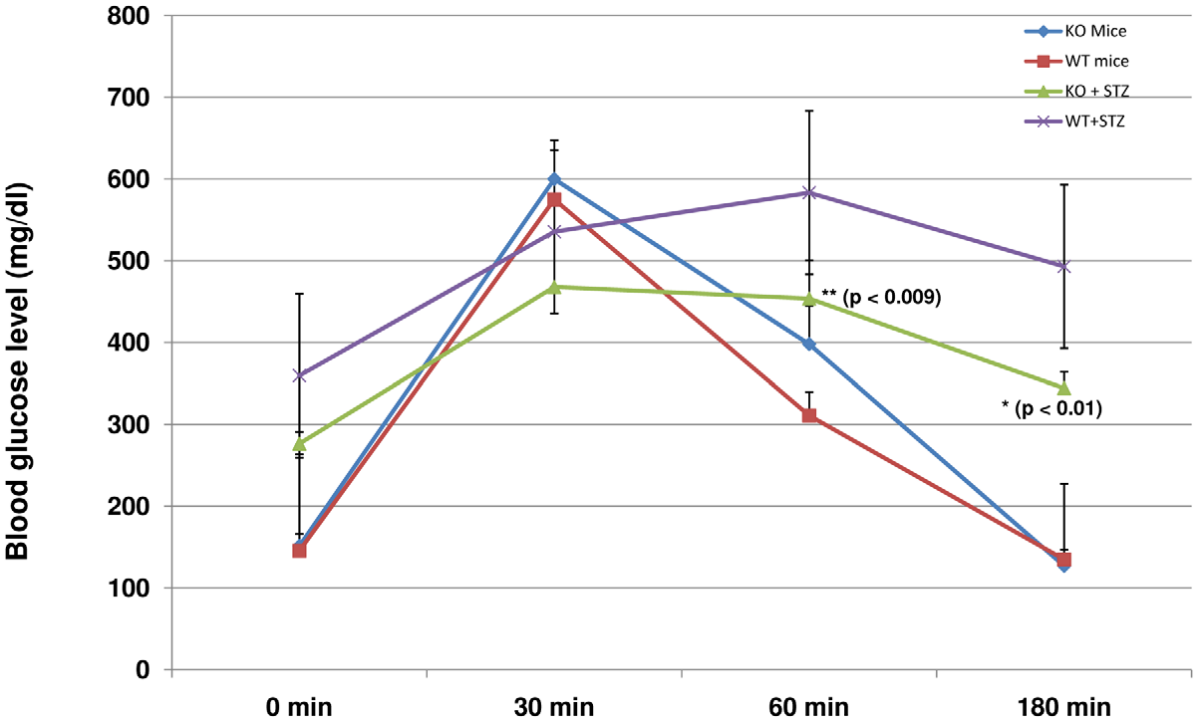
Glucose tolerance test in control or streptozotocin (STZ)-treated OX^−/−^ and C57BL/6 mice. The graph shows blood glucose levels in control or STZ-treated OX^−/−^ and C57BL/6 mice 0, 30, 60 and 180 min after intra-peritoneal glucose challenge (3 g/kg body weight, given intra-peritoneally). Blood glucose levels of control OX^−/−^ and C57BL/6 mice were similar at time 0, 30, 60, 180 min. Note that blood glucose level of STZ-induced diabetic OX^−/−^ mice was slightly but not significantly lower than that of diabetic C57BL/6 mice at 0 min. Diabetic OX^−/−^ mice displayed a significantly (*p*<0.01) lower blood glucose levels compared to those of diabetic wild type at 60 and 180 min after glucose load.

We observed that OX_1_R co-localizes with INS in pancreatic beta cell and it is up-regulated in diabetes. Control OX^−/−^ and C57BL/6 and STZ-treated OX^−/−^ and C57BL/6 mice were challenged with intra-peritoneal glucose load to examine whether the orexin/OX_1_R system has a role to play in glucose metabolism. Blood glucose levels of control OX^−/−^ and C57BL/6 mice were similar at time 0, 30, 60, 180 min. In contrast, blood glucose level of STZ-induced diabetic OX^−/−^ mice was slightly but not significantly lower than that of diabetic C57BL/6 mice at 0 min. Diabetic OX^−/−^ mice displayed a significantly lower blood glucose levels compared to those of diabetic wild type at 60 and 180 min after glucose challenge. The efficacy of glucose handling by STZ-treated OX^−/−^ mice approached that of control OX^−/−^ and C57BL/6 mice (Fig. 10).

## Discussion

### OX_1_R in pancreatic nerves of normal and diabetic rats

The observations of this study showed that OX_1_R is present in the intrinsic neuronal ganglion and nerve fibers innervating the pancreas of both normal and diabetic Wistar rats. There was no difference between the pattern of distribution of OX_1_R-postive nerves in normal and diabetic Wistar rat pancreas. The presence of OX_1_R-immunopositive nerves confirms the result of previous studies from our laboratory [21]. OX_1_R expression has been reported in the submucosal and myenteric plexuses of the guinea-pig and rat ileum [12]. Orexins/orexin receptors have also been implicated in several functions in the alimentary tract. These biological functions include local neuronal excitations, gut motility, blood flow and absorption and secretion by alimentary epithelium [10]. OX_1_R-positive nerves may release orexins to act as a conduit in the implementation of various biological functions in the pancreas and in other organs. Previous studies have shown that Orexin-A stimulates pancreatic fluid and protein release [31]. This orexin-induced increase in pancreatic exocrine secretion is mediated through the vagus, the cranial nerve that innervates the pancreas. This indicates that branches of the vagus supplying the pancreas and other abdominal organs may contain OX_1_R.

### OX_1_R in the islet cells of normal and diabetic rats

The present study and those reported previously [21] from our laboratory confirm that the number of OX_1_R-positive cells in the islet of Langerhans increased significantly in diabetic rats compared to normal. The reason(s) for the increase in the number of OX_1_R-immunoreactive cells in the islet of diabetic rats is not clear. A possible reason could be the low intracellular glucose level observed in type 1 DM. The loss of insulin in diabetic rats means less glucose will be transported into most cells, thus up-regulation OX_1_R expression. Several reports have shown that orexin-positive neurons [32] as well as OX_1_R and OX_2_R [33] are activated during fasting. The number of OX_1_R-positive cells in both the central and peripheral regions of the islets of Langerhans increased after the onset of diabetes. OX_1_R-positive cells are not the only cells that increase after the onset of diabetes, for example the number of GLU-immunoreactive cells increased significantly in diabetes [22]. It is worth noting that OX_1_R was observed only in pancreatic islet cells as well as in nerves. OX_1_R expression was not seen in the parenchymal cells of the exocrine pancreas. It is not clear why OX_1_R is not observed in the parenchymal cells of the exocrine pancreas. The fact that OX_1_R expression was observed in both neurons and pancreatic endocrine cells of the islet of Langerhans is not surprising because there is similarity in the origin of neurons and islet cells. In fact, some studies have shown that the endocrine pancreas mature along a pathway similar to that of neuron [34]. OX_1_R may therefore be confined to cells of neuronal origin.

It was interesting to observe OX_1_R expression in the islet of GK rats, an animal model of type 2 diabetes. The expression of OX_1_R in the pancreas of GK rats was higher than those in the pancreas of normal Wistar rat but similar to that seen in the pancreas of diabetic Wistar rats. This observation probably points to a common denominator that induces increased OX_1_R expression in the pancreatic islet of type 1 diabetic rats.

### Co-localization of OX_1_R with INS- and GLU in the pancreatic islets of normal and diabetic Wistar and GK rats

Several pancreatic beta cells of normal Wistar rats contained OX_1_R. In diabetic Wistar rat pancreas, the number of INS-positive cells expressing OX_1_R decreased with a concomitant increase in the number of OX_1_R-positive cells. It is well known that the number of INS-positive cells is decreased in diabetes [35]. The co-localization of OX_1_R INS in the pancreas of normal Wistar rats indicates a functional role for OX_1_R in the regulation of insulin metabolism and consequently that of glucose. GLU-immunoreactive cells expressed OX_1_R in the islets of normal and diabetic rats. It is not clear why the number of INS-producing pancreatic beta cell containing OX_1_R decreased after the onset of diabetes even though there is an increase in the number of OX_1_R-positive cells. A possible reason could be an existence and proliferation of a subpopulation of pancreatic beta cell than contain only OX_1_R. Another reason could be the absence of INS in old and newly formed OX_1_R-positive cells.

The co-localization of OX_1_R and INS is further confirmed by the fact that a similar pattern was seen in the islet of GK rats, an animal model of type 2 diabetes, where hyperglycaemia is much milder. All of these support a functional role of OX_1_R and the orexinergic system in the regulation of carbohydrate metabolism.

This is the first report on the co-localisation of INS and GLU and OX_1_R. The number of GLU-positive cells expressing OX_1_R increased significantly after the onset of type 1 diabetes. Although, OX_1_R is present in the pancreatic islets of GK rats, a model of type 2 diabetes, the degree of co-localization with GLU was minimal compared to that STZ-induced diabetic rat (a model of type 1 diabetes). This may be due to the fact that the hyperglycaemia is much milder in GK compared to STZ-treated Wistar rats. The co-localization of OX_1_R with INS and GLU suggests a pivotal role for INS and GLU in the biological actions of orexins. Moreover, orexins evoked the release of INS [36] and GLU [37] from normal pancreatic islets. This orexin-induced INS release may be due to GLU action. GLU as we know, stimulates INS secretion [38].

**Figure 11:**
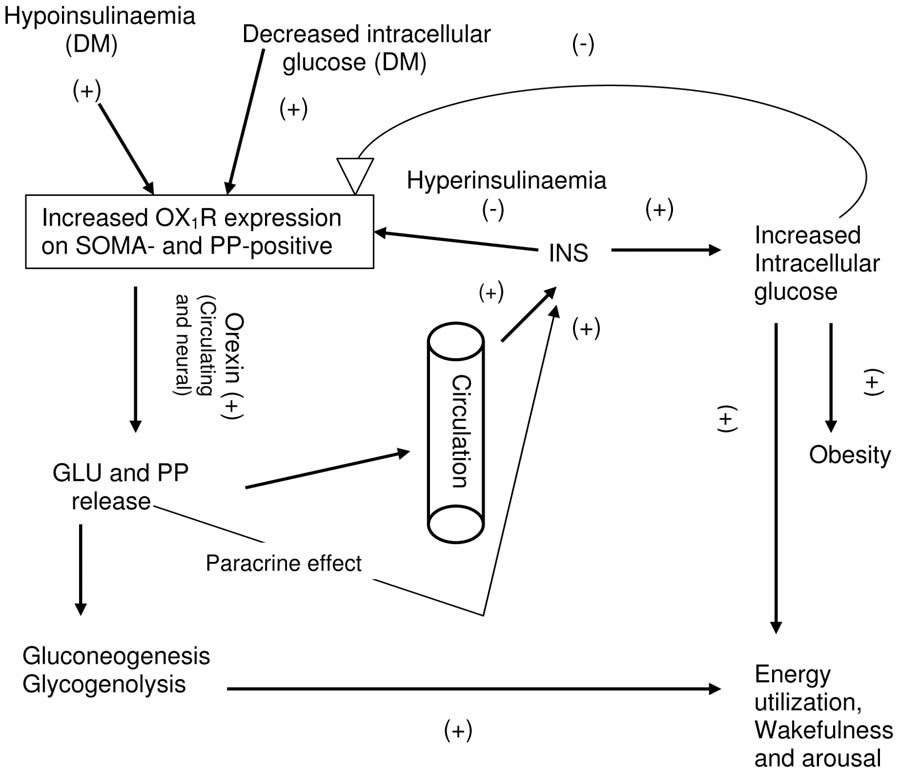
Schematic diagram of putative mechanism of orexin-induced increase in food and water intake, wakefulness and arousal. Diabetes mellitus, with the resulting decrease in intracellular glucose, leads to increased expression of OX_1_R in pancreatic islet cells such as glucagon (GLU) and pancreatic polypeptide (PP). All of these, in combination with circulating and neural-derived orexins stimulate GLU and PP release. GLU induces gluconeogenesis and glycogenolysis resulting in energy utilization, wakefulness and arousal. Moreover, GLU and PP from the circulation and via paracrine effect may stimulate insulin (INS) release resulting in increased intracellular glucose, and energy utilization. DM = diabetes mellitus; OX_1_R = orexin-1 receptor; INS = insulin; GLU = glucagon; PP = pancreatic polypeptide. (+) = stimulate; (−) = inhibit.

OX_1_R was also upregulated in PP-positive islets cells after the onset of diabetes (result not shown). The reason for the increase in the expression of OX_1_R in pancreatic polypeptide (PP)-immunoreactive cells is unknown. Since OX_1_R is present in PP-positive cells, orexin may stimulate the release of PP, which may in turn enhance appetite. It has been shown that centrally administered PP elicits food intake via NPY receptors [39]. The hypothetical relationship between pancreatic hormones, orexins, obesity, energy utilization, wakefulness/arousal is depicted in figure 11.

### Time course of diabetes-induced changes in OX_1_R expression and quantitative analysis

This study showed a larger increase in OX_1_R expression in the pancreatic islets of rats with long-term DM compared to those with short-term diabetes. The reason for this observation is not clear, however, it may be due to the irreparable damage done to pancreatic tissue due to persistent hyperglycaemia and other inductors of oxidative stress. Oxidative stress is a major cause of late complications of DM and may be more pronounced in the pancreas of rats with long and established DM [40]. This may in turn contribute to the high tissue level of OX_1_R protein observed in the pancreas of rats with long-term diabetes.

### OX_1_R and markers of apoptosis

Cleaved caspase 3, indicative of an activated enzyme, is a critical step in the apoptosis pathway. Expression of active caspase 3 was discernible in the cells of the peripheral regions of the islets of OX^−/−^ and C57BL/6 mice and normal Wistar rats. The number of caspase 3-positive islet cells was significantly fewer in OX^−/−^ mice compared to C57BL/6 mice. It is worth noting that OX^−/−^ mice have significantly fewer OX_1_R compared to wild type. It does appear that the number of OX_1_R-positive cells is directly proportional to that of caspase 3. The use of OX^−/−^ mice shows that the fewer the number of OX1R-immunoreactive cells, the lower the number of caspase 3-positive cells becomes.

In contrast, the number of OX_1_R- and caspase 3-postive cells increased significantly after the onset of STZ-induced diabetes in Wistar rats. A significant number of pancreatic islet cells of this animal model contain both OX_1_R- and caspase 3. This shows a link, *albeit* in diabetic rat pancreas, between orexin and apoptotic pathways.

A link between OX_1_R and apoptosis has been described by Voisin et al, [27], Laburthe et al [28] and El Firar et al [29]. They showed that orexins induce apoptosis via OX_1_R in cancer cell lines.

### OX_1_R and the apoptotic marker, PARP in pancreatic islets of control or STZ-treated OX^−/−^ and C57BL/6 mice

Western blot technique showed the expression of PARP in the pancreas of both orexin knockout and wild type mice. The tissue level of PARP decreased after the induction of experimental diabetes in orexin-deficient mice. It is not clear why PARP was well expressed in the pancreas of normal wild type and knockout mice at least when they were not subjected to any toxins that could possibly induce oxidative stress and therefore apoptosis.

The pancreas of orexin knockout mice contained a small quantity of OX_1_R compared to the pancreas of the wild type. However, the tissue level OX_1_R increased after the onset of diabetes in the mutant, but not in control mice. The reason for the increase in not known, but it may be due to the modulation of the orexinergic system by STZ, especially in the absence of orexin in the mutant mice. The increased expression of OX_1_R after the onset of diabetes in the mutant mice was accompanied by a decrease in the expression of PARP when compared to control, non-diabetic mutant mice and control as well as diabetic wild type. The reason for this inverse relationship is unclear but it may be due to the absence of orexin, a major trigger of apoptosis.

### Glucose handling in control OX^−/−^ and C57BL/6 and STZ-treated OX^−/−^ and C57BL/6 mice

We showed for the first time that STZ-induced diabetic orexin knockout mice shows a more efficient glucose handling compared to control OX^−/−^ and C57BL/6 and STZ-treated C57BL/6 mice. Since STZ causes oxidative stress and apoptosis [40], [41], it is of great interest to observe a reduced PARP expression in the mutant mice. It is possible that STZ induces pancreatic beta cell apoptosis via the orexin pathway. So if orexin is missing, as in the mutant mice, then less apoptosis will occur and more beta cell will survive after the administration of STZ. The mutant mice injected with STZ will therefore handle glucose more effectively as observed in our study.

### Conclusion

The number of cells expressing OX_1_R is increased in pancreatic islets after the onset of diabetes. The longer the duration of diabetes the more intense the OX_1_R expression becomes. OX_1_R-positive islet cells co-localizes with INS and GLU in Wistar rats and with cleaved caspase 3 after the onset of diabetes in Wistar rats treated with STZ. The expression of OX_1_R and caspase 3 is significantly lower in the pancreas of orexin knockout mice compared to wild type and Wistar rats.

Orexin knockout mice treated with STZ have the ability to handle glucose load more effectively that control OX^−/−^ and C57BL/6 and STZ-treated C57BL/6 mice. STZ requires the orexinergic system to cause severe apoptosis that can consequently lead to an overt diabetes.
